# Stage IVb thymic carcinoma: patients with lymph node metastases have better prognoses than those with hematogenous metastases

**DOI:** 10.1186/s12885-017-3228-2

**Published:** 2017-03-27

**Authors:** Yu Yang, Xing-Wen Fan, Hong-Bing Wang, Yin Xu, Dou-Dou Li, Kai-Liang Wu

**Affiliations:** 1 Department of Radiation Oncology, Fudan University Shanghai Cancer Center, Shanghai, 200032 China; 2 Department of Oncology, Shanghai Medical College, Fudan University, Shanghai, 200032 China

**Keywords:** Thymic carcinoma, Staging classification, Lymph node metastasis, Hematogenous metastasis

## Abstract

**Background:**

This study aimed to analyze the pattern of lymphogenous and hematogenous metastases in patients with stage IVb thymic carcinomas and identify prognostic factors for their survivals.

**Methods:**

Between September 1978 and October 2014, 68 patients with pathologically confirmed stage IVb thymic carcinomas were treated at Fudan University Shanghai Cancer Center. Forty-three patients had lymph node involvement without distant metastases, and the remaining 25 patients had hematogenous metastases. Clinical-pathological characteristics, including age, sex, histologic subtype, tumor size, metastasis, treatment modalities, such as surgical resection, radiotherapy, and chemotherapy, and clinical outcomes, such as overall survival (OS) and progression free survival (PFS), were analyzed.

**Results:**

The median follow-up time was 22 months (range, 1–126 months). The median OS of all patients with stage IVb thymic carcinomas was 30 months, and the 5-year overall survival rate was 25.1%. The median PFS was 11 months, and the 5-year PFS was 17.9%. Stage IVb patients with lymph node involvement had a better survival than those with distant metastasis (40 vs. 20 months, *p* = 0.002). Patients with myasthenia gravis had a worse prognosis (*p* = 0.033). Multivariate analysis identified metastatic status as an independent prognostic factor for OS in patients with stage IVb thymic carcinomas.

**Conclusions:**

Patients with lymph node involvement had a better survival than those with distant metastases. Much work remains to investigate the prognosis of patients with stage IVb thymic carcinomas and to explore different treatment strategies for patients with lymph node involvement and distant metastases.

## Background

Thymic carcinoma is a relatively rare mediastinal neoplasm characterized by extensive local invasion and distant metastasis. Thymic carcinoma accounts for 5% of all thymic malignancies [1], and it has a 5-year survival of 30–50% [2–5]. No specific staging system has been developed for thymic carcinoma because of its rarity. Thus far, we have used the Masaoka-Koga staging system, which is the most widely accepted system currently and an excellent predictor of prognosis for thymoma patients [6, 7]. The Masaoka-Koga staging system classifies both lymphogenous and hematogenous metastases as stage IVb tumors. Thymic carcinoma often metastasizes lymphogenously or hematogenously, while thymoma does not [8–10].

In general, thymic carcinomas are more invasive and metastatic, have a higher risk of relapse, and lead to shortened survival, compared to thymomas, which are indolent tumors that tend to recur locally rather than metastasize [4, 8]. Thymic carcinoma prognostic factors include Masaoka-Koga stage [11, 12], resection status [11, 13], pathological subtype [1], and innominate vessel invasion [8]. Most patients with thymic carcinoma present with advanced disease at initial diagnosis. While advanced thymic carcinoma portends a worse outcome, prognostic information is needed to aid clinicians in selecting patients with metastatic thymic carcinomas who are more likely to obtain a good quality of life and long-term survival. Nevertheless, previous studies have mainly highlighted clinical approaches with curative intent, paying little attention to the subgroup of patients with stage IVb thymic carcinomas undergoing palliative treatment. It is necessary to analyze the patterns of lymphogenous and hematogenous metastases of thymic carcinoma and to investigate whether prognosis is influenced by these metastases.

Here, we analyzed the difference in survival outcomes between patients with lymphogenous metastasis and those with hematogenous metastasis of thymic carcinoma, and we identified prognostic factors for survival of patients with stage IVb thymic carcinomas.

## Methods

### Patients

We retrospectively analyzed the data from 68 patients with stage IVb thymic carcinomas who had been treated at Fudan University Shanghai Cancer Center between September 1978 and October 2014. This study was approved by the Institutional Review Board of Fudan University Shanghai Cancer Center and conducted in accordance with the Declaration of Helsinki. The eligibility criteria were as follows. (1) Patients were diagnosed with histologically confirmed thymic carcinoma based on the World Health Organization (WHO) classification of thymic epithelial tumors [14]. The classifications were reclassified according to WHO by experienced pathologists. (2) Patient tumors that exhibited lymphogenous or hematogenous metastasis were staged as stage IVb thymic carcinoma according to the Masaoka-Koga staging system [15], a modification of Masaoka staging system. (3) Patients had to have complete follow-up data.

There were 68 patients with pathologically confirmed stage IVb thymic carcinoma, including 43 patients with lymphogenous metastasis and 25 patients with hematogenous metastasis, and the data from these patients were evaluated in this study.

Thymic carcinoma metastasis involved two groups of lymph nodes, the anterior (perithymic) lymph nodes (N1) and deep intrathoracic or cervical nodes (N2), consistent with the proposal of the Thymic Domain of the Staging and Prognostic Factors Committee (TD-SPFC) [16]. Lymphogenous or hematogenous metastasis was determined based on radiological findings of computed tomography, magnetic resonance imaging, or positron emission tomography and confirmed by surgical resection or percutaneous biopsy.

### Statistical analysis and definition of analysis factors

Tumors were considered completely resected if all margins were microscopically negative. Tumors were considered incompletely resected if the margins were microscopically positive or if a subtotal or partial resection was performed. Tumors that disseminated were regarded as incompletely resected, even if all nodules were resected macroscopically. Patients who received biopsy alone were regarded as inoperable.

Data collected included age, sex, histologic subtype, tumor size, metastasis, treatment modalities, such as surgical resection, radiotherapy, and chemotherapy, and clinical outcomes, such as overall survival (OS) and progression free survival (PFS).

The primary outcomes were OS and PFS. OS was defined as the time interval from the date of surgery, or the day of primary treatment if no surgery had been performed, to the date of death from any cause or the date when last known to be alive. PFS was defined as the time interval from the date of surgery, or the day of primary treatment if no surgery had been performed, to the date of recurrence, disease progression, or the date of last follow-up. Statistical survival analysis was performed using SPSS (version 17.0, SPSS Inc., Chicago, IL, USA). Survival curves were constructed using the Kaplan-Meier method and compared using the log-rank test. Cox regression analysis was performed for multivariate analysis. All *p* values were 2-sided, and statistical significance was set at *p* ≤ 0.05.

## Results

### Patient characteristics

Patient clinical and pathologic characteristics are summarized in Table 1. Among 68 patients diagnosed with stage IVb thymic carcinomas, there were 54 men and 14 women, with a mean age of 52 years (range, 16–86 years). There was a slight preponderance of male patients (79.4%). The most common histologic type of thymic carcinoma was squamous cell carcinoma (*n* = 46). The cases of thymic squamous cell carcinomas were graded (G1, G2, G3). Tumor grading showed that 13.04% of the cases were G1, 23.91% were G2, and 63.04% were G3. At presentation 2 patients presented with myasthenia gravis (MG) and 16 patients (23.5%) were asymptomatic. The presenting symptoms included 24 cases with chest pain, 11 cases with cough, 7 cases with cervical mass, 5 cases with superior vena cava syndrome, 4 cases with dyspnea and one case with hoarseness. The mean tumor size was 9 cm (range, 3 cm–23 cm) in maximum dimension.

**Table 1:** Univariate Analysis of Progression Free Survival and Overall Survival in 68 Stage IVb Thymic Carcinomas

	Number (%)	Progression free survival *P* value	Overall survival *P* value
Age at diagnosis (years)	52 (16–86)	0.157	0.135
< 52	33 (48.5%)		
≥ 52	35 (51.5%)		
Sex		0.647	0.753
Male	54 (79.4%)		
Female	14 (20.6%)		
Histology		0.135	0.454
Squamous cell carcinoma	46 (67.6%)		
Neuroendocrine carcinoma	6 (8.8%)		
Adenocarcinoma	4 (5.9%)		
Mucoepidermoid carcinoma	1 (1.5%)		
Clear cell carcinoma	1 (1.5%)		
Uncategorized	10 (14.7%)		
Grading		0.341	0.188
G1	6 (13.1%)		
G2	11 (23.9%)		
G3	29 (63.0%)		
Myasthenia gravis		0.523	0.033
Yes	2 (2.9%)		
No	66 (97.1%)		
Tumor size	9 (3–23)cm	0.662	0.464
< 9	14 (20.6%)		
≥ 9	17 (25.0%)		
Unknown	37 (54.4%)		
Metastasis		0.054	0.002
Lymphogenous	43 (63.2%)		
Hematogenous	25 (36.8%)		
Lymph node metastasis		0.281	0.361
Anterior lymph nodes	10 (14.7%)		
Deep lymph nodes	33 (48.5%)		
Hematogenous metastasis		0.423	0.461
Lung	11 (16.2%)		
Bone	4 (5.9%)		
Liver	3 (4.4%)		
Adrenal gland	1 (1.5%)		
Multiple organ	6 (8.8%)		
Surgical Resection		0.055	0.224
Complete resection	3 (4.4%)		
Incomplete resection	28 (41.2%)		
Biopsy only	37 (54.4%)		

### Lymph node metastasis

A total of 43 patients had lymph node involvement without distant metastasis. Among these patients, 10 patients had involvement of N1 nodes and 33 patients had involvement of N2 nodes. In the N1 group, the mean number of dissected lymph nodes was 8.8 (range, 2 to 22), and the mean percentage of positive lymph nodes was 31.8%. In the N2 group, the mean number of dissected lymph nodes was 7.4 (range, 2 to 15), and the mean percentage of positive lymph nodes was 50.39%. Most patients with N2 nodes involvement also had N1 involvement, while 11 cases developed skip metastases.

### Hematogenous metastasis

Twenty-five patients had distant metastatic disease. Among these patients, 11 patients had lung metastases, 4 patients had bone metastases, 3 patients had liver metastases, 1 patient had adrenal metastasis, and 6 patients had multiple organ metastases at presentation. Of the 6 patients with multiple organ metastases, 2 patients had lung and liver metastases, 2 patients had lung and bone metastases, and 2 patients had liver and bone metastases. In addition, there were 11 patients with both lymph node and hematogenous metastases.

### Treatment

Among the 68 patients with stage IVb thymic carcinomas, only 31 (45.6%) underwent surgical resections because surgical resection was difficult to achieve. Surgical resection was complete in 3 patients, 28 patients received an incomplete resection, and 37 patients (54.4%) were inoperable. Of the 31 patients who underwent surgical resections, 18 patients received post-operative radiotherapy, and 6 patients received adjuvant chemoradiotherapy.

Twenty-one of 43 patients with lymph node involvement underwent surgical resection (complete resection in 3 patients and incomplete resection in 18 patients). Twelve patients received post-operative radiotherapy, 6 patients received adjuvant chemoradiotherapy, one patient received neoadjuvant radiotherapy and 2 patients received adjuvant chemotherapy.

Ten of 25 patients with distant metastasis underwent surgical resection. All of the patients received an incomplete resection. Six patients received post-operative radiotherapy, one patient received adjuvant chemotherapy and 3 patients received symptomatic and supportive therapy. The distant metastatic sites of 10 patients who underwent surgical resections were lung or bone metastasis.

Among the 37 patients without operation, 8 patients received chemoradiotherapy, 15 patients received radiotherapy followed by chemotherapy, 2 patients received radiotherapy, 10 patients received chemotherapy and 2 patients received symptomatic and supportive therapy.

Of the 22 inoperable patients with lymph node involvement, 7 patients received chemoradiotherapy, 7 patients received radiotherapy followed by chemotherapy, 2 patients received radiotherapy, 5 patients received chemotherapy and one patient received symptomatic and supportive therapy.

Of the 15 inoperable patients with distant metastasis, one patient received chemoradiotherapy, 8 patients received radiotherapy followed by chemotherapy, 5 patients received chemotherapy and one patient received symptomatic and supportive therapy.

Among the 48 patients who received radiotherapy, 12 patients received two-dimensional techniques and 36 patients received 3D conformal radiation or intensity modulated radiation therapy (IMRT). The mean radiation dose delivered was 60Gy (range, 50-66Gy) with standard fractionation.

The most frequently used regimens in our study were CAP (cyclophosphamide + doxorubicin + cisplatin) and EP (etoposide + cisplatin), and both regimens had acceptable toxicity.

### Survival and prognostic factors

The median follow-up time was 22 months (range, 1–126 months). Forty-one of 68 patients with stage IVb thymic carcinomas died of the cancer itself or from tumor-related complications. The median OS of all patients with stage IVb thymic carcinomas was 30 months (range, 4–126 months), and the 5-year overall survival rate was 25.1%. The median PFS was 11 months (range, 1–116 months), and the 5-year PFS was 17.9%.

**Fig. 1:**
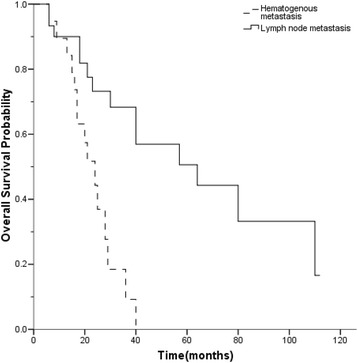
The overall survival curves of stage IVb thymic carcinoma according to metastases

**Fig. 2:**
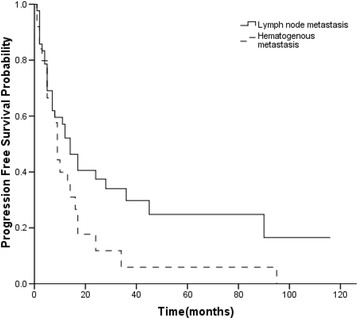
The progression free survival curves of stage IVb thymic carcinoma according to metastases

By univariate analysis, stage IVb patients with lymph node involvement had a better survival than those with distant metastasis, (median OS 40 vs. 20 months, *p =* 0.002, Fig. 1). After 11 patients with both lymph node and hematogenous metastases were excluded, patients with lymph node involvement still had a better survival than those with hematogenous metastases (*p* = 0.027). The same statistically significant difference was observed between lymphogenous and hematogenous metastases in 37 patients without operation (*p =* 0.005). Patients with MG had a worse prognosis (*p =* 0.033). However, patient variables such as age, sex, tumor size, histologic subtype and surgery resection did not show a statistical correlation with survival (Table 1). Univariate analysis showed no association of all factors with PFS. However, patients with lymph node metastasis had tended to have a better PFS (Fig. 2). The median PFS for patients with lymph node metastasis and distant metastasis were 12 and 8 months, respectively. None of the differences in PFS (*p* = 0.281) and OS (*p* = 0.361) reached statistical significance between patients with N1 nodes involvement and N2 nodes involvement. However, patients with N1 nodes involvement had tended to have a better PFS. There was no statistical difference in OS between patients with or without skip metastases (*p* = 0.759). Moreover, there were no significant differences in OS (*p* = 0.461) and PFS (*p* = 0.423) based on different metastatic sites. Patients with multiple organ metastases did not have a worse OS than patients with single organ metastasis (*p* = 0.125). There was no statistical difference in OS for patients who received an incomplete resection when compared with patients with biopsy (*p* = 0.918).

Multivariate analysis of the three variables (metastatic status, surgery and MG) showed that only the metastatic status was independent prognostic factors for OS (*p =* 0.005, Table 2).

**Table 2:** Multivariate Analysis of Prognostic Factors Associated with Overall Survival in 68 Stage IVb Thymic Carcinomas

Parameter	Hazard ratio (95% confidence interval)	*P* value
Myasthenia gravis		
Yes vs. no	3.807 (0.465, 31.153)	0.213
Surgery		
Yes vs. no	1.055 (0.766, 1.453)	0.744
Metastasis		
Lymphogenous vs. hematogenous	2.580 (1.341, 4.967)	0.005

## Discussion

Thymic carcinoma is a very rare tumor. Because of its rarity and the absence of prospective data, there is no specific stage classification for thymic carcinoma. We thus far have used the Masaoka-Koga staging system that was created for thymoma. With an increasing number of patients who have been diagnosed with thymic carcinoma, we found that thymic carcinomas frequently develop lymphogenous or hematogenous metastasis. One-third of thymic carcinoma patients reportedly had lymph node involvement or distant metastasis in other series [17, 18], and our data matched these findings. In general, the Masaoka-Koga staging system, which focuses on the tumor itself, classifies lymph node metastasis as stage IVb together with distant metastasis. Our study indicated that distant metastasis was an independent prognostic factor for poor OS, and patients with lymph node metastasis alone had prolonged survival. This data agreed with the results of previous studies [2, 19].

We analyzed the pattern and frequency of lymphogenous metastasis and concluded that the anterior lymph nodes were the primary lymph nodes for thymic carcinoma, with subsequent progression to the intrathoracic lymph nodes, and then the extrathoracic lymph nodes. If there was lymph node involvement, it was located in the anterior mediastinum in approximately 74.4% of patients with thymic carcinoma. However, thymic carcinoma frequently (33.3%) developed skip metastases to intrathoracic lymph nodes excluding the anterior mediastinal lymph nodes (*n* = 3), or scalene or supraclavicular lymph nodes (*n* = 8). Our study showed no statistical difference in OS between patients with or without skip metastases. Moreover, thymic carcinoma metastasized easily to lymph nodes, with invasion to neighboring organs and a large primary tumor size, as reported previously [10].

Whether the presence of a positive lymph node is a negative prognostic factor for thymic carcinoma remains controversial. Some studies have shown the prognostic value of positive lymph nodes [10, 20, 21], while other studies found no difference in survival based on their presence [2]. Park et al. reported that dissection of more than 10 lymph nodes is required to predict prognosis accurately [22]. Patients with anterior mediastinal lymph node involvement should have a better prognosis, considering that anterior mediastinal lymph nodes can be removed en bloc with the tumor in an extended thymectomy. However, in our study, the locations of lymph node metastases did not affect OS. We considered that the small size of these subgroups may have undermined the ability to assess statistically significant differences.

Many studies have confirmed that patients with thymic carcinomas who underwent a complete resection have a better prognosis than those receiving either incomplete resections or no surgery. [2, 5, 21, 23, 24] Hishida et al. analyzed 306 patients and found that stage IVb patients who underwent complete resections of loco-regional nodal and pulmonary metastases had relatively favorable survival rates (5-year OS: 60%) [11]. However, in advanced thymic carcinoma, complete resection is difficult to achieve without damaging major organs. As our study focused on stage IVb thymic carcinoma, complete resection may have been hampered in these patients due to direct invasion of adjacent tissues or metastatic spread. Thus, only 3 patients received a complete resection. Two of these patients received postoperation radiotherapy followed by chemotherapy, and the other one received chemoradiotherapy. All of the patients remain alive without progression. Whether patients with advanced thymic carcinomas could benefit from incomplete resection remains controversial [21, 25]. Hishida et al. found that maximal debulking surgery might confer a survival benefit and be worth evaluating for patients with advanced thymic tumors deemed difficult to completely resect [11], while Liu et al. failed to show an advantage from debulking surgery for thymic carcinoma [26]. Yano et al. even suggested that Masaoka stage IVb thymic carcinoma should be considered inoperable [19]. There was no OS benefit from incomplete resection in our study for stage IVb patients when compared with patients diagnosed with inoperable tumors.

The distant metastatic site was more common in lung (*n* = 11), and the following sites were bone (*n* = 4), liver (*n* = 3) and adrenal gland (*n* = 1). Considering most patients with distant metastasis are difficult to receive surgical resection, we analyzed the difference between lymph node metastasis and distant metastasis in 37 patients without operation. Our results show that the survival outcomes of patients with distant metastasis (*n* = 15) are worse than those of patients with lymph node metastasis (*n* = 22). Based on prognosis, the Masaoka-Koga staging system definition of stage IVb thymic carcinoma as a tumor that has either lymphogenously or hematogenously metastasized is imprecise.

Our study has limitations typical of the retrospective nature of the data collection and heterogeneity of treatments. However, this kind of bias is intrinsic to the study strategy and thus unavoidable. Furthermore, because of the low incidence and indolent behavior of thymic carcinoma, few patients have been analyzed. Thus, large-scale prospective trials and collaborative efforts will be needed to provide sufficient prognostic information to establish an appropriate staging system and to predict survival and recurrence for patients with this rare disease.

## Conclusions

In conclusion, our results demonstrate that patients with stage IVb lymph node metastasis may have a better survival than patients with distant metastases. Much work remains to clarify the classification scheme and investigate the prognosis of patients with thymic carcinoma.

## Abbreviations

IMRT: Intensity modulated radiation therapy
MG: Myasthenia gravis
OS: Overall survival
PFS: Progression free survival
TD-SPFC: The Thymic Domain of the Staging and Prognostic Factors Committee
WHO: World Health Organization
